# Variable Effects of Acoustic Trauma on Behavioral and Neural Correlates of Tinnitus In Individual Animals

**DOI:** 10.3389/fnbeh.2016.00207

**Published:** 2016-10-25

**Authors:** Ryan J. Longenecker, Alexander V. Galazyuk

**Affiliations:** Department of Anatomy and Neurobiology, Northeast Ohio Medical UniversityRootstown, OH, USA

**Keywords:** gap-induced prepulse inhibition of the acoustic startle reflex, prepulse audiometry, single unit recording, inferior colliculus, tinnitus, hearing loss

## Abstract

The etiology of tinnitus is known to be diverse in the human population. An appropriate animal model of tinnitus should incorporate this pathological diversity. Previous studies evaluating the effect of acoustic over exposure (AOE) have found that animals typically display increased spontaneous firing rates and bursting activity of auditory neurons, which often has been linked to behavioral evidence of tinnitus. However, only a subset of studies directly associated these neural correlates to individual animals. Furthermore, the vast majority of tinnitus studies were conducted on anesthetized animals. The goal of this study was to test for a possible relationship between tinnitus, hearing loss, hyperactivity and bursting activity in the auditory system of individual unanesthetized animals following AOE. Sixteen mice were unilaterally exposed to 116 dB SPL narrowband noise (centered at 12.5 kHz) for 1 h under ketamine/xylazine anesthesia. Gap-induced prepulse inhibition of the acoustic startle reflex (GPIAS) was used to assess behavioral evidence of tinnitus whereas hearing performance was evaluated by measurements of auditory brainstem response (ABR) thresholds and prepulse inhibition PPI audiometry. Following behavioral assessments, single neuron firing activity was recorded from the inferior colliculus (IC) of four awake animals and compared to recordings from four unexposed controls. We found that AOE increased spontaneous activity in all mice tested, independently of tinnitus behavior or severity of threshold shifts. Bursting activity did not increase in two animals identified as tinnitus positive (T+), but did so in a tinnitus negative (T−) animal with severe hearing loss (SHL). Hyperactivity does not appear to be a reliable biomarker of tinnitus. Our data suggest that multidisciplinary assessments on individual animals following AOE could offer a powerful experimental tool to investigate mechanisms of tinnitus.

## Introduction

Tinnitus, the perception of sound in the absence of an external sound source, is often developed after acoustic over exposure (AOE; Hoffman and Reed, 2004; Møller, 2011; Baguley et al., 2013). Animal studies have shown that AOE leads to cochlear damage and subsequent threshold shifts (Liberman and Kiang, 1978; Kujawa and Liberman, 2009). Following this damage, the central auditory system increases its gain to compensate for the reduced sensorineural input from the cochlea, which can lead to tinnitus (Salvi et al., 2000; Schaette and McAlpine, 2011; Auerbach et al., 2014). In most clinical cases, there is a strong correlation between hearing loss and tinnitus (Lockwood et al., 2002), but interestingly, this is not always true, as some patients with clinically normal thresholds have tinnitus (Weisz et al., 2006; Job et al., 2007). Just as in humans (Hall et al., 2016), the extent of peripheral damage and central plasticity in individual animals of the AOE model differs greatly, leading to a heterogeneous population of hearing loss (HL) and/or tinnitus pathology (Longenecker and Galazyuk, 2011; Singer et al., 2013; Hickox and Liberman, 2014; Knipper et al., 2015).

Ever since the first animal model of tinnitus (Jastreboff et al., 1988), research has focused on being able to separate animals into T+ and T− groups to reach conclusions concerning the underlying pathology related to AOE. Following AOE animals typically demonstrate increased spontaneous firing, bursting, and neural synchrony at multiple levels of the central auditory system (see Roberts et al., 2010; Kaltenbach, 2011; Wang et al., 2011). Recordings form anesthetized animals have revealed coincidence of hyperactivity and increased burst firing in the cochlear nucleus (Kaltenbach and Afman, 2000; Chang et al., 2002; Brozoski and Bauer, 2005; Finlayson and Kaltenbach, 2009; Pilati et al., 2012), inferior colliculus (IC; Wang et al., 1996; Ma et al., 2006; Bauer et al., 2008; Coomber et al., 2014), medial geniculate body (Kalappa et al., 2014), and auditory cortex (Syka and Rybalko, 2000; Noreña and Eggermont, 2003). It is thought that hyperactivity could manifest as the phantom sound of tinnitus (Gerken, 1996; Salvi et al., 2000; Eggermont and Roberts, 2004). In contrast to many of these pioneer works, recent studies have found that hyperactivity is not always linked to tinnitus because only a fraction of animals in these studies exhibited behavioral signs of tinnitus after AOE (Ropp et al., 2014: Coomber et al., 2014). Similarly, increased bursting activity was elevated in the IC of all noise-exposed guinea pigs, but there was no difference found between T+ and T− groups (Coomber et al., 2014). However, excluding hyperactivity and increased bursting as neural correlates of tinnitus would be inappropriate because there is no compelling evidence that these abnormalities are absent in T+ animals. It is possible that more than one, or a specific combination of these neural correlates is required for tinnitus percept.

The vast majority of tinnitus studies have been conducted on anesthetized animals, which might alter experimental results and lead to erroneous conclusions. Ketamine, perhaps the most used anesthetic drug in animals, is known to affect many molecular targets which include: disrupting N-methyl-D-aspartate (NMDA) receptors, Ih currents, nicotinic acetyl-choline channels, nitric oxide, α-amino-3-hydroxy-5-methylisoxazole-4-propionic acid (AMPA) receptors, and metabotropic glutamate receptors (mGluRs; Sleigh et al., 2014). Ketamine also has long-term effects on suppression of many immediate early genes, NMDA receptor phosphorylation, and reduced astrocyte and microglial function (Sleigh et al., 2014). While awake recordings can be challenging, the data gathered from the relatively unaltered nervous system might elucidate new information about the neural correlates of tinnitus.

In the present study, we investigate the effects of AOE on behavioral hearing thresholds, auditory brainstem response (ABR) thresholds, and behavioral signs of tinnitus assessed by gap-induced prepulse inhibition of the acoustic startle reflex (GPIAS) on 16 CBA/CaJ mice. To investigate several suspected neural correlates of tinnitus in detail, we performed single unit recordings in 4 mice out of the 16 behaviorally tested mice. To eliminate a possible effect of anesthesia, all single unit recordings were performed in awake animals. We found that each of the four animals studied in depth demonstrated a unique phenotype following near-identical AOE. All of these mice showed some degree of increase spontaneous activity, while only one showed increased burst firing. While results should be considered in light of the small sample size presented here, future tinnitus studies might benefit from studying individual animals to elucidate the neural mechanisms of tinnitus.

## Materials and Methods

### Subjects

A total of 20 male CBA/CBJ mice were used. Sixteen mice were used for the experimental group which included behavioral testing pre exposure and 3–4 months post exposure. Four mice from the experimental group were randomly selected for single unit recordings. The remaining four mice were also used for single unit recordings as controls (age-matched, without AOE). Mice were obtained from Jackson Laboratories, Bar Harbor, ME, USA and were approximately 12 weeks old with a mean weight of 27.5 g at the beginning of behavioral testing. Mice were housed in pairs within a colony room with a 12-h light–dark cycle at 25°C. Procedures used in this study were approved by the Institutional Animal Care and Use Committee at the Northeast Ohio Medical University.

### Acoustic Trauma

Sixteen mice were anesthetized with an intraperitioneal injection of a ketamine/xylazine mixture (100/10 mg/kg). Mice were at least 5 months old at the time of exposure. An additional injection (50% of the initial dose) was given intramuscularly 30 min after the initial injection. Mice were exposed to a one octave band noise centered at 12.5 kHz (~8–17 kHz) unilaterally for 1 h. This noise was generated using a waveform generator (Wavetek model 395), amplified (Sherwood RX-4109) to 116 dB Sound Pressure Level (SPL), and played through a loudspeaker (Fostex FT17H). The output of the loudspeaker was calibrated with a 0.25-inch microphone (Brüel and Kjaer, 4135) and found to be ±4 dB between 10 and 60 kHz. The left external ear canal was obstructed with a cotton plug and a Kwik-Sil silicone elastomer plug (World Precision Instruments), a manipulation which typically reduces sound levels by 30–50 dB SPL (Turner et al., 2006; Ropp et al., 2014).

### Auditory Brainstem Response (ABR) Thresholds

Mice were anesthetized with ketamine/xylazine. ABR thresholds were obtained by presenting tone bursts at 4, 12.5, 16, 20, 25 and 31.5 kHz at increasing sound intensities ranging from 10–80 dB SPL in 10 dB steps. Tones were 5 ms in duration, with 0.5 ms rise/fall time and delivered at the rate of 50/s. ABR thresholds were obtained before, directly following, and 3 months after acoustic trauma. Sterile stainless-steel electrodes were placed subdermally, one behind the right pinna of the sound exposed ear and the other along the vertex. The unexposed ear was obstructed with a cotton plug. Evoked potentials were averaged over 300 repetitions. These potentials were amplified (Dagan 2400A preamplifier), filtered (100–3,000 Hz bandpass), digitized (HEKA Elektronik), and stored on a computer hard drive. Thresholds, the smallest sound amplitude that evoked a visible ABR, were determined by visually examining the averaged ABR waveforms in response to every sound frequency presented at different sound levels.

### Behavioral Assessments of Tinnitus and Hearing Loss

Prior to exposure, the 16 experimental mice were behaviorally tested with GPIAS and prepulse inhibition (PPI) to obtain baseline values for gap detection and hearing thresholds. Mice were assessed for tinnitus/threshold shifts 3 months after exposure. Four mice were randomly selected to highlight the importance of individual differences.

#### Acoustic Startle Hardware/Software

The equipment used to collect all acoustic startle reflex (ASR) data has been described in detail previously (Longenecker and Galazyuk, 2012). Briefly, commercial hardware/software equipment from Kinder Scientific, Inc. Poway, CA, USA was used. Each behavioral testing station was lined with anechoic foam to prevent sound reflection and wave cancelling sound echoes (Sonex foam from Pinta Acoustics, Minneapolis, MN, USA). Mice restrainers were open walled to allow for maximum sound penetration (Figure 3 in Longenecker and Galazyuk, 2012). Background sound levels within each testing chamber were calibrated with a 0.25-inch microphone (Brüel and Kjaer 4135) attached to a measuring amplifier (Brüel and Kjaer 2525) and found to be less than 40 dB SPL between 4 and 60 kHz. Startle waveforms were recorded using load-cell platforms which measure actual force changes during an animal’s startle. Each load cell was calibrated with a 100 g weight which corresponds to 1 newton of force. Offline waveform analysis converted these forces into animals’ center of mass displacement (in mm; Grimsley et al., 2015).

#### Startle Waveform Identification and Measurement

All waveforms collected during testing sessions were analyzed offline using a recently developed automatic method of startle waveform identification via a template matching paradigm (Grimsley et al., 2015). In this study, we used high-speed video recordings (1,000 frames/s) to visualize animal startles in order to identify stereotyped waveforms associated with a startle. This allowed us to develop custom software which automatically separates data into either startles or non-startle-related movements. Based on this separation, we have only included trials that resulted in successful startle responses in our data analysis. We also used a mathematical approach to normalize startle response magnitudes of individual animals to their body mass (Grimsley et al., 2015). This mathematical conversion allows legitimate comparisons between animals of different mass.

#### GPIAS for Tinnitus Assessment

The ability of mice to detect a gap of silence preceding the startle stimulus was determined using a startle stimulus presented alone (startle only: SO) and a startle stimulus paired with a gap (Gap + startle: GAP) both embedded into continuous background noise. The gap had a 20 ms duration and 1 ms rise/fall time. Background for all these trials was presented as a narrow band (1/3 octave) noise centered at six different frequencies (8, 10, 12.5, 16, 20 and 25 kHz). This background noise level was constant (65 dB SPL) throughout the session. The startle stimulus was presented at 105 dB SPL (white noise, 1 ms rise/fall, 20 ms duration). The gap was presented 100 ms before (onset to onset) the startle stimulus. The testing session started with an acclimation period lasting 3 min. Immediately afterwards, animals received five SO trials in order to habituate their startle responses to a steady state level. For each of six background frequencies, we presented five SO trials and five GAP trials. The SO and GAP trials were pseudo-randomized. The inter-trial intervals were also pseudo-randomized between 7 and 15 s. After we completed testing all six background frequencies, the entire session was repeated two more times. Thus, during this testing for each background frequency we obtained 15 data points for both the SO and GAP trials.

Tinnitus was classified in the same way as in previous studies (Longenecker and Galazyuk, 2011; Dehmel et al., 2012; Coomber et al., 2014). A two-way repeated measures analysis of variance (ANOVA) was applied to the behavioral data to determine whether there were any significant changes (*p* < 0.05) in gap detection levels before vs. 3 months after exposure. A Least Significant Difference (LSD) *post hoc* test was used to determine the specific frequency of any deficits. Importantly, this allowed each animal to act as its own control. Mice that exhibited a significant reduction in gap detection ability at one or two background frequencies after noise exposure were categorized as “tinnitus positive” (T+) animals while those that did not were assigned to a “tinnitus negative” (T−) group.

#### PPI Audiometry for Hearing Assessments

PPI audiometry was described in detail previously (Longenecker et al., 2016), and heavily relied on the advanced startle waveform analysis mentioned above (Grimsley et al., 2015). Hearing thresholds assessed by PPI audiometry were conducted at the same experimental epochs as GPIAS tests to ensure behavioral data were comparable and relevant. Testing sessions contained two types of stimuli. The SO stimulus was identical to the GPIAS tests except it was presented at 100 dB SPL. The second stimulus type consisted of a congruent startle stimulus preceded by a prepulse. Prepulse stimuli were 20 ms pure tones with a 1 ms rise/fall time presented at six different frequencies (4, 12.5, 16, 20, 25 and 31.5 kHz) 100 ms before the startle stimulus. Prepulses were presented pseudo-randomized by intensity (10–80 dB SPL, 10 dB step) for each sound frequency (each frequency was one block of testing; six blocks total). Each frequency/intensity combination was presented 39 times. SOs were pseudo-randomly mixed throughout each testing session. In each session, the magnitudes of the SOs were compared to the magnitudes of startles preceded by prepulses with various frequencies and intensities. A significant reduction in the magnitude of the startle response by the prepulse compared to the SO was defined as the prepulse detection threshold.

Identifying this threshold involved several steps. First, we examined the distribution of the raw startle magnitudes. Startle magnitude is known to be quite variable in mice, and indeed the startle magnitude typically was strongly positively skewed. This positive skew could allow aberrantly high values to obscure reliable changes in startle magnitude across stimulus parameters. Furthermore, a normal distribution of magnitudes is an assumption underlying the method for threshold determination that we use here. Thus we transformed the data (Tukey, 1977). A square root transform was found to best generate a normal distribution of startle responses within each trial type, as assessed using the Anderson-Darling test. This standard statistical transform reduces skew by enhancing the lesser and reducing the larger startle magnitudes. Second, for each animal, the transformed SO data was bootstrapped to determine 95% confidence intervals for the SO response magnitudes. Then we calculated medians of transformed magnitudes for each frequency and intensity, as the median value is a better measure of central tendency than the mean for skewed distributions. For each frequency, a detection function was calculated by fitting a cubic spline from the median transformed SO magnitude through the median transformed magnitudes for prepulses presented at various intensities. Detection threshold was defined as the sound level at which the fitted detection function crossed the lower 95% confidence interval.

Each animal was classified as having hearing loss (HL), severe hearing loss (SHL), or no hearing loss (NoHL) based on the number of frequencies at which PPI thresholds were elevated. HL was defined as at least a 30 dB threshold increase in at least two frequencies, while SHL was similarly defined, but with more than two frequencies affected. These classifications can be seen in Figure 2.

### Electrophysiological Recordings

#### Surgery

Four mice from the control and four mice from the sound exposed groups were used for extracellular recordings. Each mouse was anesthetized using isoflurane (1.5–2.0%) prior to surgery. A midline incision of the skin over the cranium was made. The tissue overlying the skull then was removed and a small metal rod was glued to the skull using glass ionomer cement (3 M ESPE, Germany). Following 2 days of recovery, each mouse was trained to stay inside a small plastic tube, to be used as a holding device during recording sessions. The metal rod on the head of the mouse was secured to a small holder designed to restrain the head of the animal without causing distress, while the ears were unobstructed for free-field acoustic stimulation.

#### Extracellular IC Recordings

Recordings were made from both the ipsi- and contra-lateral IC (compared to the side of exposure) in awake mice inside a single-walled sound attenuating chamber (Industrial Acoustics Company, Inc., North Aurora, IL, USA). Throughout the recording session (2–3 h), the animal was offered water periodically and monitored for signs of discomfort. After a recording session, the exposed skull was covered with sterile bone wax and the animal was returned to its holding cage. Experiments were conducted every day for 6 days after which the animal was sacrificed with an IP injection of FatalPlus. No sedative drugs were used during recording sessions. If the animal showed any signs of discomfort, the recording session was terminated and the mouse was returned to its cage.

Recording electrodes were inserted through a small ( 50 μm) hole drilled in the skull and dura overlying the IC. Extracellular single-unit recordings were made with quartz glass micropipettes (10–20 MΩ impedance, 2–3 μm tip) filled with 0.5 M sodium acetate. The electrode was positioned into the drilled hole by means of a precision (1 μm) digital micromanipulator using a surgical microscope (Leica MZ9.5). The relative position of each electrode was monitored from the readouts of digital micrometers using a common reference point on the skull.

Extracellular recordings were limited to the central nucleus of the IC based on the depth of recordings. Vertical advancement of the electrode was made by a precision piezoelectric microdrive (Model 660, KOPF Instruments) from outside the sound-attenuating chamber. Recorded action potentials were amplified (Dagan 2400A preamplifier), monitored audio-visually on a digital oscilloscope (DL1640, YOKOGAWA), digitized and then stored on a computer hard drive using EPC-10 digital interface and PULSE software from HEKA Elektronik at a bandwidth of 100 kHz.

The search stimulus consisted of a train of tone bursts (20 ms duration, 5–50 kHz, 5 kHz step). This train was repeated while the recording electrode was advanced in 3 μm steps. To ensure that the sample of neurons we recorded was unbiased based on frequency tuning, the characteristic frequency of recorded neurons was assessed manually by presenting tone pips 100 ms in duration using a wide range of sound frequencies (4–50 kHz, 2 kHz step) and different sound levels (20, 40, 60 dB SPL). The spontaneous rate (SR) of neuronal firing was assessed during a 40 s recording (without stimulus). No sound-evoked recordings are reported.

## Results

### Behavioral Signs of Tinnitus After Sound Exposure

**Figure 1:**
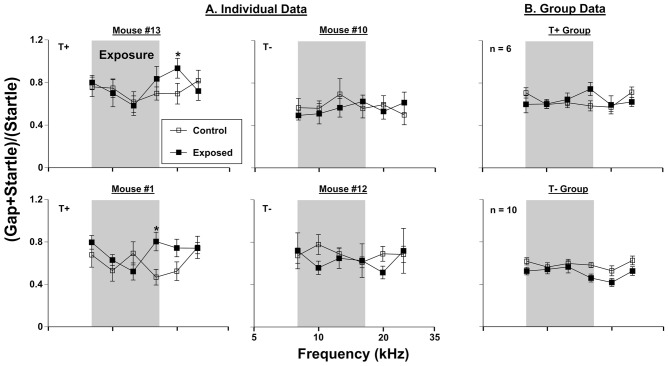
**Gap-induced prepulse inhibition of the acoustic startle reflex (GPIAS) assessment for tinnitus. (A)** Four individual mice were tested before (control) and 3 months (exposed 3M) after sound exposure. **(B)** Group GPIAS data for T+ mice (*n* = 6) and T− mice (*n* = 10), in the same conditions as **(A)**. The narrow band exposure stimulus is represented by a gray box. Data are represented by ratio means and standard errors. Tinnitus was identified as a significant (*) difference between control and exposed conditions. Tinnitus positive and negative mice are labeled as (T+) and (T−), respectively.

Behavioral assessments using GPIAS methodology were conducted on 16 mice before vs. 3 months after sound exposure. Six of 16 mice developed significant gap detection deficits at frequencies between 12.5 and 25 kHz. Since gap detection deficits have been associated with tinnitus (see Galzyuk and Hébert, 2015), these mice constituted the T+ group. The remaining 10 mice did not show gap detection deficits and they were considered as T−. Data from two representative mice from each group are shown in Figure 1. Comparison of the gap detection performance before vs. 3 months after exposure with a repeated measures ANOVA indicated a significant effect of AOE in mouse #13 (*F*_(1, 75)_ = 666.11, *p* < 0.001) and mouse #1 (*F*_(1, 61)_ = 661.73, *p* < 0.001). A LSD *post hoc* test revealed a frequency-specific deficit at 20 kHz for mouse #13 (*p* = 0.05) and 16 kHz for mouse #1 (*p* = 0.012). Two representative mice from T− group (#10 and #12) did show significant overall differences in gap detection performance before vs. 3 months after exposure (mouse #10 (*F*_(1, 57)_ = 326.40, *p* > 0.001); mouse #12 (*F*_(1, 50)_ = 435.22, *p* > 0.001), but LSD* post hoc* tests did not reveal any frequency specific differences (Figure 1). When data from all six T+ mice were averaged, a significant effect of exposure was seen (*F*_(1, 30)_ = 1562.12, *p* < 0.000). However, LSD* post hoc* tests did not determine any specific tinnitus frequencies, although the deficits at 16 kHz were close to significance (*p* = 0.053). The 10 animals determined to be T− did not show any significant gap detection deficits after exposure, but interestingly, did tend (not significant) to demonstrate improved gap detection performance at frequencies above the sound exposure.

### Effect of Sound Exposure on Hearing Performance

**Figure 2:**
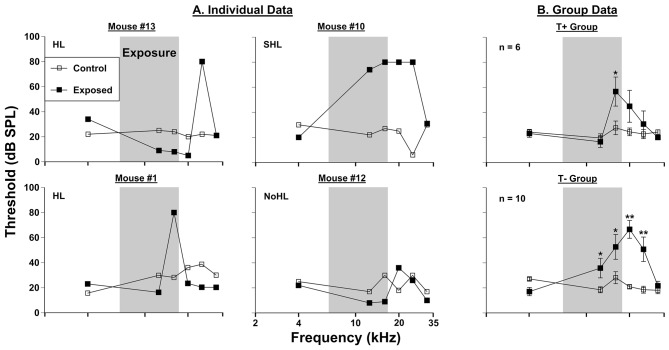
**Auditory thresholds assessed via prepulse inhibition (PPI) audiometry. (A)** Four individual mice were tested before (control) and 3 months (exposed 3M) after sound exposure. **(B)** Group PPI data for T+ mice (*n* = 6) and T− mice (*n* = 10; from Figure 1B), in the same conditions as **(A)**. The narrow band exposure stimulus is represented by a gray box. Each animal was classified as having hearing loss (HL), severe hearing loss (SHL), or no hearing loss (NoHL) based on the number of frequencies at which PPI thresholds were elevated. Significant threshold shifts indicated with (*) at *p* = 0.05 level or (**) at *p* = 0.001 level.

Hearing thresholds assessed by PPI audiometry revealed inter-animal differences in the severity of threshold shifts in AOE mice (Figure 2), and each animal was classified based on the criteria explained above in “PPI Audiometry for Hearing Assessments” Section. Threshold elevations were seen in 13 of the 16 exposed mice. Six of these 16 mice showed narrowband frequency deficits. Examples of this type of deficit are shown in mouse #13 (at 25 kHz) and mouse #1 (at 16 kHz) in Figure 2. Seven out of 16 mice exhibited severe deficits spanning a wider frequency range. A representative mouse from this population (mouse #10) showed elevated thresholds from 12.5 kHz to 25 kHz (Figure 2). Finally, 3 out of 16 mice did not show PPI audiometric threshold changes after noise exposure (Figure 2, representative mouse #12). When animals were grouped together by T+ and T− classifications (as in Figure 1), differences in threshold elevations were observed (Figure 2). The T+ group demonstrated increased thresholds following sound exposure (*F*_(1, 30)_ = 316.56, *p* < 0.001), LSD* post hoc* tests showed a significant threshold shift only at 16 kHz (*p* = 0.01). The T− group by comparison demonstrated further exaggerated thresholds after sound exposure (*F*_(1, 54)_ = 385.37, *p* < 0.001). This group of mice, which comprised more animals with the “SHL” classification, showed significant threshold increases at 12.5 kHz (*p* = 0.035), 16 kHz (*p* = 0.003), 20 kHz (*p* < 0.001) and 25 kHz (*p* < 0.001).

**Figure 3:**
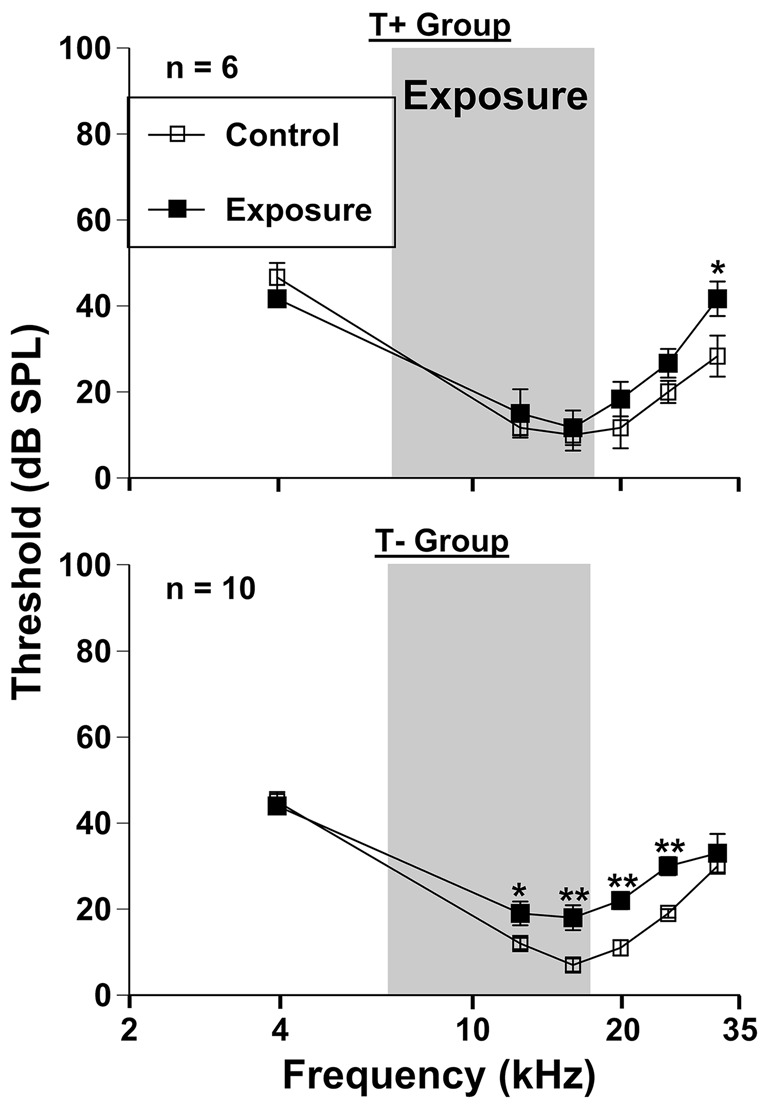
**Auditory thresholds assessed via auditory brainstem response (ABR) grouped by T +** **(*n* = 6) and T− (*n* = 10) animals (from Figure 1B).** Mice were tested before (control) and 3 months (exposed 3M) after sound exposure. The narrow band exposure stimulus is represented by a gray box.

ABRs were collected to serve as a control for PPI hearing assessments following sound exposure (Figure 3), however these measures were compared extensively in our previous work (Longenecker et al., 2016). Thresholds of the T+ group nearly returned to baseline levels 3 months following exposure, with an average increase across frequencies of 6.11 dB SPL (*F*_(1, 30)_ = 478.47, *p* < 0.001). *Post hoc* LSD tests revealed that only 31.5 kHz significantly differed from control levels (*p* = 0.021), with a 13.33 dB SPL shift. These results contrasted ABR threshold shifts from the T− group, which as mentioned previously, encompassed more animals determined to have SHL measure by PPI. This group showed an average threshold increase of 7.33 dB SPL across frequencies after exposure (*F*_(1, 54)_ = 1366.24, *p* < 0.001). Specific deficits were seen at 12.5 kHz (*p* = 0.037), 16 kHz (*p* = 0.001), 20 kHz (*p* = 0.001), and 25 kHz (*p* = 0.001). Not surprisingly, the range of frequencies showing a deficit with ABR (Figure 3) closely followed the deficits seen by PPI audiometry (Figure 2), although ABR deficits were not nearly as pronounced.

### Increased Spontaneous Activity Following Sound Exposure

**Figure 4:**
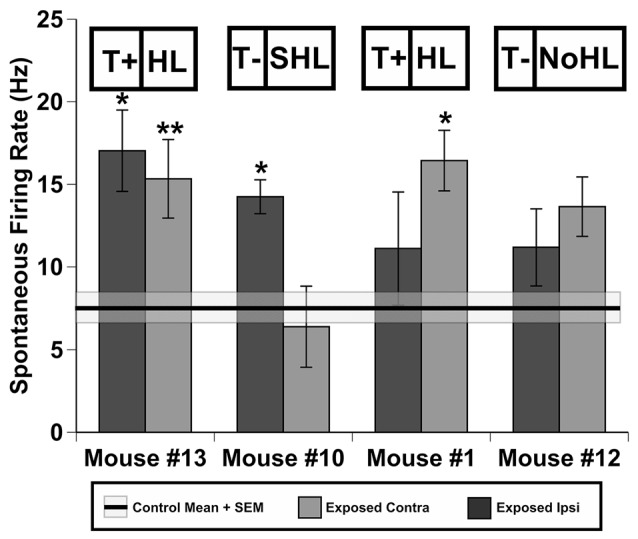
**Spontaneous firing of inferior colliculus (IC) neurons in four individual mice after sound exposure**. Data represents means and standard errors of spontaneous rate (SR)s of contra- and ipsi-lateral ICs (relatively to the exposed ear) for each mouse. Signifcant differences (*0.05, **0.001) between each IC of exposed mice and averaged values across four control (unexposed) animals (black line = mean, shaded region = std. error). Tinnitus and hearing loss abbreviations are taken from Figures 1, 2 respectively.

To determine how spontaneous firing rates of IC neurons changed for each behavioral AOE outcome single unit recordings were conducted in awake mice. Behavioral AOE outcomes included classifications for T+ and T− groups as well as the severity of hearing loss (based on number of elevated thresholds; 0 = NoHL; 1–2 = HL; 3–6 = SHL). Spontaneous activity from 118 neurons recorded in four control (unexposed) mice was compared to the activity of 384 neurons in the four exposed mice described above. In the exposed mice, the ipsi- and contra-lateral ICs were considered separately in an attempt to differentiate the possible effects of unilateral sound exposure. A one-way ANOVA was used to compare spontaneous firing rates of IC neurons between control and exposed (both ipsi and contra exposed IC’s) mice (*F*_(500, 8)_ = 5.34, *p* < 0.001; Figure 4). The Tukey HSD* post hoc* tests revealed significant increases in neural firing rates following sound trauma in the following IC’s: #13contra (*p* < 0.001), #13ipsi (*p* = 0.012), #10contra (*p* = 0.043), #1ipsi (*p* = 0.030). Although not all statistically significant, seven out of eight ICs demonstrated increased mean spontaneous rates. Interestingly, mouse #10’s ipsilateral IC demonstrated a non-significant slight decrease in activity compared to control rates (*p* = 1.00), however, the ipsi IC had significantly higher neural firing rates than the contralateral IC (*t*_(64)_ = 6.23, *p* < 0.001).

### Effect of Sound Exposure on Burst Firing in IC Neurons

To determine the effect of sound exposure on bursting activity in IC neurons, the spontaneous firing of 118 neurons from control (unexposed) mice and 384 neurons from exposed mice was analyzed and compared. These are the same pool of neurons that have been used for assessing spontaneous firing rate changes described above.

**Figure 5:**
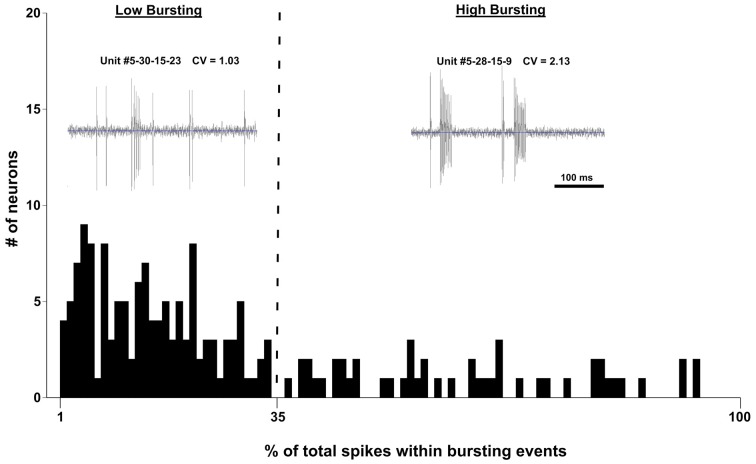
**Histogram of the percentage of spikes that occurred in bursting events of 502 IC neurons recorded from four control mice and four exposed mice.** Units were separated into Low Bursting (LB) and High Bursting (HB) units using a 35% cutoff point (vertical dashed line) which was determined statistically. Examplar units for each bursting classification are shown in the upper part of the figure. Each unit includes its coefficient of varation (CV) which represents a measure of spike regularity. Higher CV values mean that a given neuron is bursting more.

Bursting activity has been linked to tinnitus-like behavior in many studies (review by Wang et al., 2011). In our study, we adopted the following burst classifications from Bauer et al. (2008) to define a bursting event: (1) maximum allowable burst duration: 310 ms; (2) maximum ISI at burst start: 500 ms; (3) maximum within-burst ISI: 10 ms; (4) minimum interval between bursts: 50 ms; (5) minimum burst duration: 5 ms; and (6) minimum number of spikes comprising a burst: 2 ms. The distribution of burst firing neurons (Figure 5) was not bimodal as determined by the Kolmogorov-Smirnov test of normality (*D*_(178)_ = 0.176, *p* < 0.001). However, if the data were separated into low bursting and high bursting units at the 35% mark, the two divisions became normally distributed (Low Bursting (LB): *D*_(53)_ = 0.97, *p* = 0.09; High Bursting (HB): *D*_(48)_ = 0.106, *p* = 0.20; Figure 5). Neurons with no bursting (NB) were not include in this distribution. Therefore, we developed a new classification system to further separate bursting activity into three categories: high bursting, low bursting, or non-bursting based on the percentage of total spikes within each bursting event (Figure 5).

**Figure 6:**
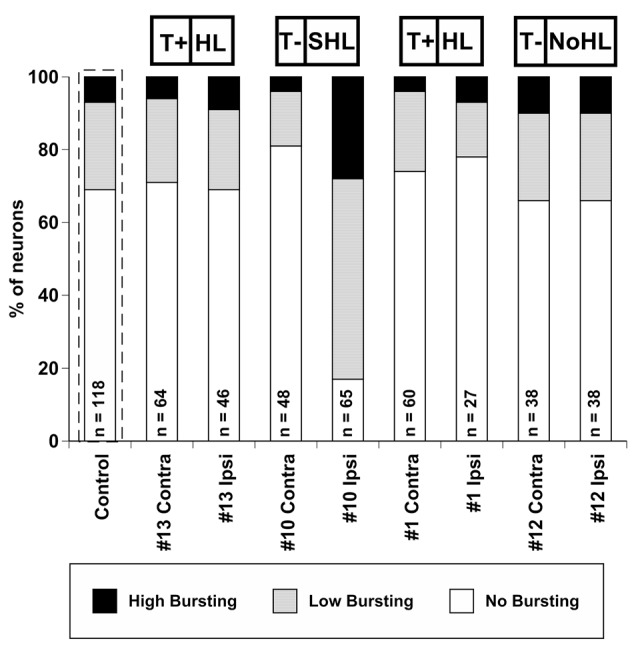
**Percent distribution of auditory neurons having different levels of bursting activity (high bursting, low bursting, and no bursting) in conta- and ipsilateral ICs of four exposed mice.** The average distribution from four control (unexposed) mice is outlined by a dashed line. The number of neurons collected is labeled for each exposed IC (or all ICs for control). Tinnitus and hearing loss abbreviations are taken from Figures 1, 2 respectively.

The percentage of units exhibiting bursting activity was plotted based on the degree of bursting activity (Figure 6). In control, unexposed mice, 31% of neurons demonstrated bursting activity (24% LB, 7% HB), while 69% of the neurons did not show bursting activity. This observation is similar to the bursting rate distributions found in sound exposed guinea pigs (Coomber et al., 2014). The percentage of bursting neurons in mouse #13, #1, and #12 were similar to the control (unexposed) distribution. Furthermore, the differences between ipsi- and contralateral IC’s were surprisingly small. In contrast, mouse #10 demonstrated a unique change in bursting activity following sound exposure. Eighty-three percentage of the neurons in the ipsilateral IC of this mouse showed some degree of bursting activity (55% LB, 28% HB). Interestingly, the contralateral IC of mouse #10 had only 19% of its neurons displaying bursting, which was the lowest value among all IC’s tested.

**Figure 7:**
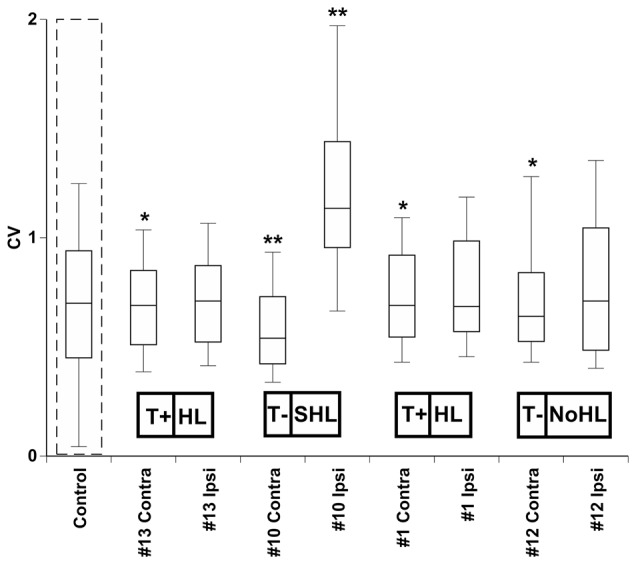
**Distribution of CVs across conta- and ipsilateral ICs of four exposed mice.** The average CV from four control (unexposed) mice is outlined by a dashed line. Significant differences between control and each exposed IC was determined by a paired *t*-test (*0.05, **0.001). Tinnitus and hearing loss abbreviations are taken from Figures 1, 2 respectively.

To quantify changes in bursting activity, we applied the coefficient of variation (CV) analysis, a statistic that is derived by normalizing the standard deviation of each unit’s ISI distribution by its mean (Ma et al., 2006; Kalappa et al., 2014). Units with high CVs had more irregular ISIs, which suggests that they had more bursting activity (Figure 7). The normal physiological range for CVs has been reported as 0.5 to 1 in cortical neurons (Christodoulou and Bugmann, 2001), however recent studies have shown values of 1.2 or higher in the dorsal cochlear nucleus (DCN) following acoustic trauma (Pilati et al., 2012). Statistical comparisons of the mean distribution of CVs across each IC of four exposed mice were tested with a Wilcoxon Signed Rank Test to evaluate whether sound exposure changed the bursting patterns in IC neurons (Figure 7). Small but significant CV decreases were observed in the contra IC of mouse #13 (*Z* = −2.67, *p* = 0.007), mouse #1 (*Z* = 2.15, *p* = 0.049) and mouse #12 (*Z* = 2.92, *p* = 0.003). A much more significant CV increase was only observed for the ipsilateral IC for mouse #10 (*Z* = −4.24, *p* < 0.001), and a large significant decrease was found in the contralateral IC for mouse #10 (*Z* = −3.06, *p* = 0.002).

## Discussion

The important finding of this study was that despite identical sound exposure parameters, behavioral and neural assessments revealed a diverse set of pathologies among mice. Behavioral evidence of tinnitus was observed in just under half of mice exposed, which was often accompanied with some degree of threshold shifts measured by PPI audiometry. The burst firing rate in animals classified as T+ was not significantly different from the control animals. The bursting activity, however, greatly increased in one animal with significant PPI threshold shifts. Increases in spontaneous activity following sound exposure were observed regardless of behavioral evidence of tinnitus or hearing loss in the small sample of mice tested.

To this point, most animal model tinnitus studies have grouped T+ and T− animals together in order to explain differences in any number of physiological factors that change after AOE. While this is a reasonable approach to make conclusions about sample populations that underwent the identical experimental manipulations, it does not lend credence to the significant variability of peripheral, central auditory, emotional, and cognitive aspects of the human tinnitus population (Langers et al., 2012). Clinically, this sort of problem might be best addressed by increased data sharing and a systematic review of all clinical studies (Hall et al., 2016). However, in animal studies both group and individual results should be included in order to parse apart the various factors that could influence tinnitus manifestation. Below, we discuss the individual results of a small sample of mice that underwent several behavioral and electrophysiological evaluations.

### Behavioral Data: Group vs. Individual Analysis

We found that 6 out of 16 mice developed GPIAS deficits consistent with behavioral evidence of tinnitus 3 months following AOE. This percentage of T+ animals fits within the range of other studies that report between 30% and 75% rates of tinnitus among animals tested (see Galzyuk and Hébert, 2015). The T+ group (*n* = 6) showed a non-significant, although close (*p* = 0.053), GPIAS deficit at 16 kHz, whereas the T− group (*n* = 10) did not show deficits at any frequencies, and in fact showed non-significant improvements in high frequency gap detection following AOE (Figure 1). It is not surprising that GPIAS results assessed in the T+ group did not show a significant deficit because the frequency of the gap detection deficit varies among mice, although usually manifests at, or above the center frequency of exposure (Longenecker and Galazyuk, 2011; Turner et al., 2012; Coomber et al., 2014; Ropp et al., 2014). Thus, 16 kHz would fall within this expected deficit range following an AOE with a 12.5 kHz centered noise. Indeed, all individual animal GPIAS deficits ranged from 16 kHz to 25 kHz, of which two examples can be seen in Figure 1.

PPI audiometry can provide behavioral thresholds in a matter of a couple hours compared with months of training for behavioral audiograms (Heffner et al., 2008; Radziwon et al., 2009). Because behavioral thresholds are considered the “gold standard” for auditory evaluations, it is important that they are used in conjunction with tinnitus assessments. With the same 16 mice tested for behavioral evidence of tinnitus, we found that thirteen showed increased behavioral thresholds when assessed by PPI audiometry 3 months after AOE. Of those 13 with increased thresholds, six showed narrowband frequency deficits restricted to one or two neighboring frequencies tested, while wide-band deficits were seen in seven mice. The animals with narrowband deficits, had PPI threshold increases within the same 16–25 kHz band as GPIAS deficits were usually seen. This finding does bring into question whether the animals with specific deficits (like mouse #1) have tinnitus. If GPIAS (Figure 1) and PPI deficits (Figure 2) match in frequency it is possible that the mouse could not hear the background noise during the GPIAS testing and thus showed a gap detection deficit at that frequency. Seven mice however showed broadband deficits, exemplified by mouse #10 (Figure 2). If the dramatic PPI audiometric threshold elevations are reflecting peripheral damage, it would imply that many of these animals could not hear well. Future experiments should identify the level of cochlear damage at which GPIAS could not be used for tinnitus assessment.

Thresholds assessed by ABRs were nearly identical for all animals before and 3 months after AOE (Figure 3), with only minor permanent threshold increases. Although this does not exclude “hidden hearing loss” as a result of deafferentation at the ribbon synapses of the cochlea (Schaette and McAlpine, 2011). ABR wave one amplitudes would better assess these changes (Kujawa and Liberman, 2009; Lin et al., 2011). In a mirror study with the same AOE parameters we found that wave one amplitudes were dramatically decreased, especially at suprathreshold levels, and more importantly, that these deficits corresponded to PPI audiometric deficits (Longenecker et al., 2016). Therefore, we expect that these animals also experience a decrease in ABR wave one amplitudes, signifying peripheral deafferentation (Kujawa and Liberman, 2015). Only small threshold shifts were seen at 31.5 kHz in the T+ group, which contrasted with the more significant deficits seen in the T− group between 12.5 and 25 kHz (Figure 3). This ABR data correlates well with PPI threshold data (Figure 2), which suggests that T− animals might have a tendency towards more wide spread peripheral and/or central damage (Roberts et al., 2012). A similar finding demonstrated that non-tinnitus rats had a higher expression of activity-regulated cytoskeletal (Arc) immediate early gene measured across the hippocampus, amygdala, and auditory cortex (Singer et al., 2013). This means more central plasticity occurred in animals with SHL, and more importantly that tinnitus generation might require a more focused peripheral/central damage.

### Relationship Between Hyperactivity and Tinnitus

AOE causes peripheral damage that gradually leads to central plastic changes, possibly leading to tinnitus in some individuals, but not others. Possible reasons for this variability are discussed below. Regardless of the behavioral variability of tinnitus, some neural correlates have consistently been seen after AOE (see Eggermont, 2016), many in the IC (see Berger and Coomber, 2015). For several decades animal studies have suggested hyperactive neuronal activity is a neural correlate of tinnitus (see review by Eggermont, 2005, 2013). Our study demonstrated hyperactivity is developed in all mice regardless of behavioral evidence of tinnitus (Figure 4). A similar finding has been reported for rats (Ropp et al., 2014) and guinea pigs in IC neurons (Coomber et al., 2014) as well as in neurons of the auditory cortex (Engineer et al., 2011). Interestingly, mouse #10 in our study demonstrated decreased spontaneous activity in the ipsilateral IC. This is particularly interesting when considering the bursting activity in this ipsilateral IC was dramatically increased compared to all other exposed ICs tested as discussed below. This might suggest that increased bursting activity in the IC could be an alternative neural coding strategy following decreased peripheral input (see Roberts et al., 2010). When combining the behavioral data with the single unit recordings, the results of this study would suggest that hyperactivity should be considered as a generalized outcome of sound exposure rather than a specific neural correlate of tinnitus. Our study also suggests that there does not seem to be a specific effect of being T+ and symmetry of neural firing in contra/ipsi ICs following AOE. While both the ipsi and contra ICs had significantly elevated neural activity for mouse #13 (T+ group), only the ipsi IC showed this effect in mouse #1 (T+ group; Figure 4). Mouse #10 which was T− and had SHL when measured with PPI audiometry, demonstrated a bias of hyperactivity skewed toward the contra IC. While the overall results of hyperactivity correlate well with previous studies on different model species (Coomber et al., 2014; Ropp et al., 2014), specific details of increased spontaneous activity laterality should be further studied before robust conclusions can be made.

### Bursting Activity and Tinnitus

Many studies have implicated increased bursting activity as one of several possible neural correlates of tinnitus (e.g., Ma et al., 2006; Bauer et al., 2008). Although estimates of bursting are somewhat dependent on distinct definitions of what constitutes a burst, our results clearly suggest that bursting activity decreases in the vast majority of exposed mice (Figures 6, 7) and does not correlate with behavioral evidence of tinnitus (Figure 1). There are several possible explanations for such discrepancy: (1) The effect of anesthesia. In our study extracellular recordings were conducted without the use of any anesthetics, whereas other studies were conducted on ketamine-anesthetized animals which is known to affect NMDA receptors, AMPA receptors, mGluRs, and most modulatory neurotransmitter systems which in turn might alter bursting activity (Sleigh et al., 2014). (2) The difference in timing after sound exposure. Studies assessing bursting activity have started physiological recordings a few days (Noreña and Eggermont, 2003; Finlayson and Kaltenbach, 2009), 2–3 months, (Ma et al., 2006; Coomber et al., 2014) up to a maximum of a 9 months (Bauer et al., 2008) after sound exposure. We began testing our mice at least 2 months following exposure. This matches previous behavioral (Turner et al., 2012; Longenecker et al., 2014) and physiological (Mulders and Robertson, 2011) timelines for tinnitus development. It is possible that bursting activity during the acute phase of tinnitus is very different from the chronic tinnitus. (3) The difference in sound exposure. In our study animals were exposed to a 12.5 kHz octave band noise for 1 h presented unilaterally at 116 dB SPL. Unfortunately, nearly every hearing loss/tinnitus study has adapted unique sound exposure parameters, leading to possible differential exposure effects (Galzyuk and Hébert, 2015). Thus, direct comparisons between studies are extremely tenuous, especially considering the difficulty of tinnitus assessment and internal confounds arising from various levels of hearing loss resulting from sound exposure. Any or all of these factors could explain differences in burst firing activity observed in this study.

A unique pattern of neuronal activity was observed in the one mouse with severe widespread threshold deficits. This was the only animal which showed decreased bursting contralaterally and significant increased bursting ipsilaterally (Figure 6). In line with this observation, the CV value and percentage of spikes within bursts were also increased in the ipsilateral IC while decreasing in the contralateral IC (Figure 7). These results appear paradoxical when considering the spontaneous firing rates mentioned above because the spontaneous firing rate contralaterally was much higher than control levels while the ipsilateral IC had somewhat decreased firing rates. This increased bursting activity without increasing the firing rate could represent an alternative neural strategy in compensating for the putative decreased peripheral signals. Further research is necessary to clarify neuronal mechanisms underlying changes in bursting activity following sound exposure.

### Different Pathologies Following Acoustic Exposure

Perhaps the most significant question that is left unanswered in both the literature and this study is why some animals develop tinnitus after sound exposure and others do not. This phenomenon has been indirectly reported in the vast majority tinnitus related publications but has not been studied systematically. For example, most clinical cases reported a strong correlation between hearing loss and tinnitus (Weisz et al., 2006), whereas other studies described tinnitus cases where tinnitus patients demonstrated no audiometric deficits (Schmuziger et al., 2006; Job et al., 2007; Langers et al., 2012). The diversity of tinnitus pathologies seen in the current study and other animal studies (Longenecker and Galazyuk, 2011; Coomber et al., 2014; Hickox and Liberman, 2014; Ropp et al., 2014) could be explained by a number of phenotypic factors including differences in individual animals in stress levels, unintentional noise exposure, differential peripheral damage, or unique animal-specific maladaptive neuroplasticity pattern caused by sound exposure.

#### Possible Causes of Phenotypic Diversity

Stress is known to play an important role in many disease states clinically, including tinnitus (Hébert and Lupien, 2007). Some literature suggests that stress is also an important factor for the etiology of tinnitus in animals (Singer et al., 2013). Many common housing and handling procedures can cause an animal’s stress levels to increase dramatically (for review see Balcombe et al., 2004). Stress is known to impair cognitive function (Arnsten, 2009), and thus it can alter the results of behavioral tests (Kaneto, 1997; Dawood et al., 2004; Graham et al., 2011). It has been shown that loud unexpected sounds can raise levels of stress-related hormones (Burow et al., 2005). Both restraint and social stress are common in laboratory animals (Stone and Quartermain, 1997; Ma et al., 2015). Simple handling of an animal, putting it in a new environment, and cage changes can also raise levels of stress in animals (Seggie and Brown, 1975; Duke et al., 2001). Interestingly, it was found that mice subjected to restraint stress had less dramatic permanent threshold shifts after noise exposure (Wang and Liberman, 2002). All animals in our study were subjected to restraint stress before exposure when they were placed into small restrainers during behavioral testing. Although all animals were tested the same number of times, the stress of restraint could vary between animals, leading to the differential pathologies we observed. Animals housed alone show much higher stress levels than animals housed in pairs or groups (Sharp et al., 2003). However, dominance struggles between pairs can cause a great deal of stress to the subordinate animal (Makinson et al., 2015). Any or all of these stressing factors could lead to a potential difference in the outcome of sound exposure, especially since it is known that sound exposure itself leads to increased levels of stress hormones (Kozlovsky et al., 2009).

Unintentional exposure to sound could occur in animal housing facilities (see Turner et al., 2007). A recent study recorded sounds from an animal care facility and found that weekday sound levels could easily reach the level of 70 dB SPL, but varied in intensity throughout the day (Liberman et al., 2014). Even if sound levels are not damaging, each animal would be subjected to different auditory experience which may lead to animal specific plastic changes over the length of a longitudinal study. CBA/CaJ mice conditioned to moderate levels of sound (81–89 dB SPL) before exposure demonstrated less permanent threshold shifts compared to mice that were just exposed (Yoshida and Liberman, 2000). Sound conditioning can also lead to permanent central changes as well (Turner et al., 2013). These changes can lead to changes in ABRs, prepulse behavioral measures, and startle reflex magnitudes (Turner and Willott, 1998). This suggests that an animal facility, if not closely monitored, could be greatly influencing an animal’s auditory experience and thus the results of sound exposure. Specific to the conclusions of this study, it is important to consider that each mouse could be in a slightly different area in reference to given unintentional sound source, so it is likely that sound levels were not even for every animal. Animals can also be unintentionally exposed during transportation to and from a lab or in the lab itself. These discrepancies could explain why certain mice develop tinnitus, hearing loss, or show the absence of such maladies.

Additionally, the actual conditions of exposure for each animal might differ slightly resulting in various degrees of damage. Such factors could include the exact animal placement in relation to the sound source, the time of day the animal was exposed (Meltser et al., 2014), the exact amount of anesthetic that is absorbed into the blood, as well as all stress-related factors listed above. Even among heterogeneous genotypes of guinea pig and inbred strain mice the variability of ABR threshold shifts after unanesthetized AOE can be dramatic (Wang et al., 2002). Similarly, ABR and ASR amplitudes were shown to be quite variable between mice after AOE in a recent tinnitus assessment study (Hickox and Liberman, 2014). Current theories of how this peripheral damage leads to the manifestation of tinnitus is still debated. However, it is known that exposure to loud sounds leads to permanent damage to cochlear nerve fibers, even without direct damage to inner or outer hair cells (Kujawa and Liberman, 2009; Lin et al., 2011). This was confirmed in human work, which suggested that tinnitus in patients with clinically normal audiograms may be correlated with a peripheral neuropathy, which is typically patient specific (Schaette and McAlpine, 2011; Gu et al., 2012). The resulting decrease of central input leads to maladaptive up regulation of firing in the lower auditory brainstem (for review see Roberts et al., 2010). A recent study has suggested that rats behaviorally positive for tinnitus demonstrated the greatest degree of ribbon synapse degeneration at the cochlear nerve terminal (Singer et al., 2013). Regardless of what peripheral damage occurs, the strongest evidence of the manifestation of the tinnitus percept is explained by peripherally-driven central auditory plasticity.

Central neural plastic changes resulting from AOE have been abundantly studied (see Eggermont, 2016) but how these changes result in tinnitus remains poorly understood. We know that the exposure increases the rate of peripheral degradation, assessed by threshold (Kujawa and Liberman, 2006) and suprathreshold (Fernandez et al., 2015) ABR measures. Further, it is known that unilateral peripheral damage, like the AOE in this study, drives central plasticity more significantly than bilateral lesions (Rubio, 2006). Here we describe common neuronal changes associated with tinnitus, hyperactivity and bursting activity in the IC in animals assumed to have some degree of unilateral peripheral deafferentation. The conclusions here match other recent studies that suggest hyperactivity is not associated with tinnitus but sound exposure in general (Coomber et al., 2014; Ropp et al., 2014). Additionally, a new finding in this work, albeit with a low sample size, advocates that bursting activity (Figures 6, 7) is more prevalent animals with the most significant auditory threshold shifts (Figure 2), but less so in T+ animals, with less significant threshold shifts (Figure 2). Although this finding requires more validation, due to the low sample size.

In agreement with our findings, it was found that the activity-regulated cytoskeletal protein, Arc was downregulated in the amygdala, hippocampus, and auditory cortex in mice with behavioral evidence of tinnitus but upregulated in animals with possible hearing loss (Singer et al., 2013). A similar finding in the cochlear nucleus of rats demonstrated that GAP-43 (a synaptic plasticity associated protein) was upregulated in rats with SHL but not in rats with tinnitus (Kraus et al., 2011). This suggests that certain neural activity might be downregulated in tinnitus animals but upregulated in animals with greater degrees of hearing loss. Human imaging studies have found neural plastic changes in many brain regions (see review by Simonetti and Oiticica, 2015). Most studies find that tinnitus patients show increased cerebral gray matter in the auditory pathways, and a decreased cerebral gray matter outside the auditory pathways (i.e., Limbic system, cerebellum, basal ganglia) in comparison to non-tinnitus controls. Although comparing changes in neuronal signatures between humans and animal models of tinnitus is difficult, future studies should work towards more relatable comparisons.

## Author Contributions

RJL: planned and conducted all experiments and manuscript production; AVG: planned experiments and manuscript production.

## Conflict of Interest Statement

The authors declare that the research was conducted in the absence of any commercial or financial relationships that could be construed as a potential conflict of interest.

## Abbreviations

AMPA: α-amino-3-hydroxy-5-methyl-4-isoxazolepropionic acid receptors
ANOVA: analysis of variance
AOE: acoustic over exposure
Arc: activity-regulated cytoskeletal immediate early gene
ASR: acoustic startle reflex
CV: coefficient of variation
GAP: Gap + Startle
GPIAS: gap-induced prepulse inhibition of the acoustic startle reflex
HB: high bursting activity
HL: Hearing Loss
IC: inferior colliculus
ISI: inter spike interval
ITI: inter trial interval
LB: low bursting activity
LSD: least significant difference *post hoc* test
mGluRs: metabotropic glutamate receptors
NB: no bursting activity
NMDA: N-methyl-D-aspartate receptors
NoHL: no hearing loss
PPI: prepulse inhibition
SHL: Severe Hearing Loss
SO: startle only
T+: tinnitus positive
T−: tinnitus negative.
